# Unlocking the Non-IgE-Mediated Pseudo-Allergic Reaction Puzzle with Mas-Related G-Protein Coupled Receptor Member X2 (MRGPRX2)

**DOI:** 10.3390/cells10051033

**Published:** 2021-04-27

**Authors:** Mukesh Kumar, Karthi Duraisamy, Billy-Kwok-Chong Chow

**Affiliations:** School of Biological Sciences, The University of Hong Kong, Pokfulam Road, Hong Kong, China; mkumar@connect.hku.hk (M.K.); h1258159@connect.hku.hk (K.D.)

**Keywords:** mast cells, allergy, drug-induced allergy, GPCR, hypersensitivity reactions, antagonists

## Abstract

Mas-related G-protein coupled receptor member X2 (MRGPRX2) is a class A GPCR expressed on mast cells. Mast cells are granulated tissue-resident cells known for host cell response, allergic response, and vascular homeostasis. Immunoglobulin E receptor (FcεRI)-mediated mast cell activation is a well-studied and recognized mechanism of allergy and hypersensitivity reactions. However, non-IgE-mediated mast cell activation is less explored and is not well recognized. After decades of uncertainty, MRGPRX2 was discovered as the receptor responsible for non-IgE-mediated mast cells activation. The puzzle of non-IgE-mediated pseudo-allergic reaction is unlocked by MRGPRX2, evidenced by a plethora of reported endogenous and exogenous MRGPRX2 agonists. MRGPRX2 is exclusively expressed on mast cells and exhibits varying affinity for many molecules such as antimicrobial host defense peptides, neuropeptides, and even US Food and Drug Administration-approved drugs. The discovery of MRGPRX2 has changed our understanding of mast cell biology and filled the missing link of the underlying mechanism of drug-induced MC degranulation and pseudo-allergic reactions. These non-canonical characteristics render MRGPRX2 an intriguing player in allergic diseases. In the present article, we reviewed the emerging role of MRGPRX2 as a non-IgE-mediated mechanism of mast cell activation in pseudo-allergic reactions. We have presented an overview of mast cells, their receptors, structural insight into MRGPRX2, MRGPRX2 agonists and antagonists, the crucial role of MRGPRX2 in pseudo-allergic reactions, current challenges, and the future research direction.

## 1. Introduction

The pseudo-allergic reaction is a hypersensitivity reaction manifested by immediate systemic responses. The symptoms are identical to anaphylaxis; however, pseudo-allergic reaction shares a different mechanism of mast cells (MCs) activation to that of anaphylaxis (Immunoglobulin E (IgE)-mediated) [1]. Pseudo-allergic reactions are non-IgE-mediated hypersensitivity reactions which are elicited by an initial dose of medication, and cause MC degranulation followed by the release of inflammatory and pro-inflammatory mediators [2]. It is worth noting that these reactions do not elicit antigen-specific immune responses but evoke the release of histamine and cytokines, activate the complement system, and lead to atypical synthesis of eicosanoids [3,4]. Previously, hypersensitivity reactions (HSRs) have been classified into four types; however, pseudo-allergic reactions are not categorized into any of these four classes [5,6]. One possibility is indistinguishable clinical signs and symptoms from anaphylaxis (IgE-mediated) [7,8] due to a lack of clear understanding of non-IgE-mediated mechanisms. The signs and symptoms of pseudo-allergic reactions are similar to IgE-mediated symptoms such as skin flushing, headache, edema, hypotension, urticaria, and bronchospasm [7,8]. Pseudo-allergic reactions attributed to two-thirds of HSRs [9]. However, the lack of systematic studies on the causes and mechanisms is the bottleneck for diagnosing and treating these reactions.

MRGPRX2 is a class A GPCR which binds with several endogenous and exogenous ligands. Previously, research on both MC degranulation and host response mechanism was focused on the IgE receptors (FcεRI)-mediated immune response. Investigating the alternative non-IgE mechanisms of MC degranulation and immune host response has gained immense interest recently. In the past 10 years, MRGPRX2 has emerged as a significant MC receptor responsible for non-IgE-mediated pseudo-allergic reactions. Accumulating evidence on MRGPRX2 has shown its critical role in drug-induced pseudo-allergic reactions, hypersensitivity reactions, and inflammatory diseases. MRGPRX2 is evolving as a versatile receptor with its diversity of ligands. More than 20 ligands (agonists or antagonists) have already been identified, and the quest for more agonists/antagonists that may solve the puzzle of non-IgE-mediated pseudo-allergic reactions is in full swing. However, the crucial pathophysiological roles of MRGPRX2 in allergic and non-allergic diseases remain poorly understood.

In the present review, we have comprehensively searched all of the relevant databases (PubMed, Science Direct, and Google Scholar) for the terms MRGPRX2, MRGPRX2 and allergy, pseudo-allergic reactions, and mast cell GPCR from 2006 to March 2021. Preclinical studies and systematic reviews were selected for review. Reference lists of selected studies were hand-searched to identify additional relevant studies. We have provided a brief introduction of mast cells, their receptors, and the diverse agonists of MRGPRX2. We have also discussed the crucial role of MRGPRX2 in pseudo-allergic reactions, antagonist development, current challenges, and future research directions in the MC–MRGPRX2 domain. More comprehensive studies and a clear understanding of MRGPRX2 pharmacology will contribute to a deeper understanding of several non-IgE-mediated pathologies and might offer new opportunities for designing novel putative treatments for several allergic and non-allergic disorders. 

### 1.1. Classification of MCs

MCs are granulated immune cells that possess secretory granules which release several inflammatory mediators such as proteases and inflammatory cytokines [10]. Among these mediators, proteases are lineage-defining characteristics and are confined to MCs [11]. MC chymase and tryptase have been reported to expressed ≈10,000-fold higher than non-MCs, which makes them the most selective features of the lineage [12,13,14].

MCs are classified into two types based on the protease content of their secretory granules [15]. The first type is called MCTC, or tryptase and chymase-expressing MCs; this type is found in connective tissues, mainly in the skin, and other tissues such as intestinal submucosa and myocardium. The second type called MCT or tryptase-expressing MCs found mainly in the lung and gut [16,17]. Interestingly, in mice, the difference between human MCTC and MCT has been correlated, where connective tissue MCs (CTMCs) resemble MCTC, and mucosal MCs (MMCs) resemble MCT [18,19]. The mice connective tissue MCs are mainly found in the skin, and mucosal MCs are located in the lung and gut. Besides the difference in the protease content of MCs, several other features have been reported, distinguishing the MC subtypes. These features include ultrastructural differences, secretory functions, receptor expression differences, and pharmacological responses [20,21,22]. For instance, MCTC expresses carboxypeptidase A (CPA3), cathepsin G (CTSG) and complement component receptors (C5AR1 and C3AR1), while MCT does not express these mediators and receptors [23]. However, MCTC’s C5AR1 and C3AR1 receptors are less prominent than other immune cells such as monocytes, macrophages, neutrophils, eosinophils and basophils [13,14]. 

Moreover, there is a difference in the MC degranulation response in both subtypes of MCs against endogenous and exogenous stimuli. Human MC-subtype MCTC and mouse MC-subtype connective tissue MCs respond to ligands such as compound 48/80, complement components C3a, and C5a [20,21]. In contrast, the other subtypes of both humans and mice, MCT and mucosal MCs, respectively, do not respond to these ligands [24,25,26]. Such distinctive MC responses remained enigmatic for decades. In 2006, a novel MC receptor, MRGPRX2, was discovered [27], which is confined to MCTC, and is absent in MCT [12]. MRGPRX2 now serves as a novel signature marker of MCTC [27] and clearly explains the distinctive MC response against a plethora of agonists. MCTCs are abundantly present in the skin tissue [28] and are involved in both local and systemic immune responses such as skin allergy, anaphylaxis, and systemic mastocytosis [10,29]. The role of MCs in allergic diseases has been extensively studied, with recent findings also indicating the possible role of MCTC/connective tissue MC subtypes of MCs [19] in vascular hemostasis, pain, itch, and host defense [27,30,31,32].

### 1.2. Mast Cell Receptors and Activation Mechanism

MCs regulate the innate and adaptive immune responses via releasing stored or de novo-synthesized bioactive and inflammatory mediators [10]. During homeostasis, the immune response remains in tight regulation and control to avoid overshooting and self-induced host cell damage. The immediate hypersensitivity reactions are manifested due to the rapid release of inflammatory mediators from activated MCs. The canonical mast cell activation IgE pathway is a well-studied mechanism where the antigen binds with IgE and interacts with the Fc epsilon RI (FcεRI) high-affinity IgE receptor. FcεRI has been considered as the primary receptor on MCs for decades, responsible for MCs’ activated clinical manifestations, and is still a prioritized research focus in MC biology. Several other classes of MC receptors operate in a parallel fashion to induce the immune response, such as inflammation, while at the same time, these receptors ensure the resolution of the host response to xenobiotics and other inflammogens. 

Moreover, the IgE MC activation mechanism is likely to be an over-simplification of real-time ongoing reactions inside the body. Several studies have reported that the immediate microenvironment surrounding the cells in their resident tissues and other receptors on MCs modify antigen-dependent MC activation [33]. Stem cell factor receptor (SCF/KIT) [34,35], TLRs [36], and several GPCRs [37] have been reported to modify MC responses elicited by aggregated FcεRI. These GPCR agonists elicit divergent pharmacological responses such as chemotaxis and adhesion to potentiation of FcεRI-mediated MC activation [33,37].

FcεRI is present on MCs’ surface and comprises three subunits, namely α, β and γ. The α subunit is the IgE binding site, the β subunit amplifies the signal, and two disulfide-linked γ subunits initiate the signals [38]. FcεRI-IgE aggregation elicits the tyrosine kinase cascade, culminating in granule exocytosis and rapid release of histamine and other proteolytic enzymes [37]. It is worth noting that IgE-mediated MC activation shows a relatively slow, enduring Ca^2+^ signal, compound exocytosis, and histamine is the major preformed mediator [39].

It is well known that only receptors that elicit degranulation can cause acute hypersensitivity reactions (HSRs) and allergic or pseudo-allergic reactions. Several GPCRs such as C5AR1, C3AR1, and recently identified MRGPRX2 can induce exocytosis, allergies, and anaphylactic responses. However, the clinical significance of complement receptors in HSRs has been extensively studied unlike the MRGPRX2, which is yet to explored [40,41]. Previously, the MCTC activation mechanism of several basic peptide molecules (such as substance P (SP)) and experimental drug compound 48/80 was not fully known. The MC activation mechanism of SP was reported through neurokinin 1 receptor (NK1R) [42] and direct activation of G proteins in the cytosol [43,44].

In 2006, for the first time, the selective expression and ligands activation mechanism of MRGPRX2 was reported [45]. GPCRs are membrane receptors expressed on plasma membrane; however, MRGPRX2 is also expressed in the intracellular sites of skin MCTC [46]. Ligands, such as SP, and compound 48/80 have shown MRGPRX2-dependent MC activity [45]. Although both NK1R and MRGPRX2 are expressed in MCTC, the SP-induced MC degranulation activity was dependent on MRGPRX2, not NK1R [46]. Additionally, the expression of MRGPRX2 is reported to be very high in MCTC (17,565 per 5 ng of total RNA) as compared to MCT (32 per 5 ng of total RNA) [45]. These findings are consistent with others where the PCR and microarray data demonstrated high transcription of MRGPRX2 in human skin MCTC, while MRGPRX2 transcription was low in lung MCT [46,47].

### 1.3. Mast Cell Mediators

**Figure 1:**
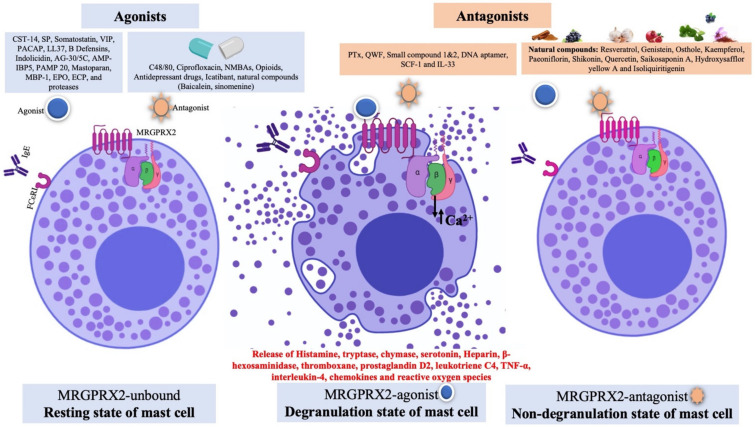
Schematic diagram showing mast cells receptors (FcεRI and MRGPRX2), MRGPRX2 ligands (agonist and antagonist) and different states of mast cells. The agonists are broadly divided into peptides and non-peptides (drugs). In an unbound state (where no ligand binds to MRGPRX2), the mast cells are in resting state. For agonists binding to MRGPRX2, the conformational changes in the receptor lead to activation of the G protein and downstream signaling pathway. This state is known as the degranulation state; the activated MRGPRX2 leads to exocytosis, MC degranulation and release of vasoactive and inflammatory mediators. However, the binding of antagonist to MRGPRX2 prevented receptor activation and inhibited MC degranulation and release of inflammatory mediators, which is known as the non-degranulation state.

MCs produce and store several bioactive mediators that are released immediately or in a delayed fashion. The MC mediators show proinflammatory and immunomodulatory activity which causes other immune cells in the body to produce more inflammatory mediators. MCs contain several preformed mediators as well as de novo mediators. The preformed mediators are histamine, serotonin, β-hexosaminidase, interleukins, tryptase, chondroitin sulfate, and heparin. The de novo mediators are prostaglandins, cysteinyl leukotriene E4, platelet activating factor, and cytokines [10] (Figure 1). Several MRGPRX2 agonists such as human β-defensins, LL-37, and angiogenic peptide-30/5C have been reported to release histamine, interleukin 8, monocyte chemoattractant proteins, macrophage inflammatory protein, tumor necrosis factor-α, and prostaglandins [48,49,50]. The MCs’ released mediators manifest their effects via binding to several GPCRs such as histamine receptor 1–4 (histamine), protease activated receptor 2 (tryptase), cysteinyl leukotriene receptor 1–2 (LTC4), and prostaglandin D2 receptor (PGD2) on end-organ targets like epithelial cells, endothelial cells, airway smooth muscle cells and nerves [51]. The typical manifestation of MC-mediated allergic reactions is wheal, flare, and pruritus, due to histamine-mediated vasodilation and increased vascular permeability. Besides histamine, MC tryptases are a key mediator of histamine-independent itch through direct activation of protease-activated receptor 2 (PAR-2) on sensory nerves [52]. Tryptase has been reported to be involved in the pathophysiology of atopic dermatitis [53,54,55].

The mechanisms of IgE- and MRGPRX2-mediated MC activation are different from each other. For example, in IgE-mediated MC activation, Ca^2+^ mobilization is relatively slow, which leads to compound exocytosis [56]. On the other hand, in MRGPRX2-mediated MC activation, Ca^2+^ mobilization is transient, which results in single granule fusion [56]. Moreover, there is a difference in the content of mediators release; histamine is the major preformed mediator in IgE, while tryptase is the major preformed mediator in MRGPRX2-mediated MC degranulation [57].

## 2. Pharmacology of MAS-Related G Protein-Coupled Receptors X 2 (MRGPRX2)

Thus far, mammals such as rodents, cattle, primates and, more recently, dogs have shown MRGPR-encoding genes [58,59]. The GPCR superfamily is classified into different classes, from A to F, according to sequence homology. Based on various conserved motifs, the MRGPR family is assigned to the rhodopsin-like class A GPCR [60,61,62]. The rhodopsin-like class A GPCR are further subdivided into four groups named A to D. MRGPR comes under the D group of class A and shares the same group as a large family of olfactory receptors, glycohormones, and purinergic receptors [60,61,62]. Still, no MRGPR subtype is declared deorphanized by NC-IUPHAR [63]. For instance, MRGPRX2, unlike any other GPCR, recognizes a wide variety of basic molecules and ligands; hence, there are numerous possible unknown ligands for this receptor. Therefore, it is still considered to be an orphan receptor regardless of possessing a plethora of ligands [64]. To deorphanize this receptor, at least two independent demonstrations of receptor–ligand pairing published in refereed papers are required [58]. Additionally, evidence of ligand binding in a given tissue via in vitro binding assays, functional assays, and anatomic data is crucial [58]. For MRGPR, at least one of the criteria mentioned is lacking; however, in a recent report in 2019 by IUPHAR/BPS, MRGPRX2 is listed as a class A orphan GPCR for which preliminary evidence for an endogenous ligand and potential disease link has been published [65].

Four MRGPRX members have been identified: MRGPRX1–X4, which shows different expression profiles and diverse functions. Briefly, MRGPRX1 is expressed mainly on small-diameter sensory dorsal root ganglia (DRG) nociceptive neurons and is involved in the perception of pain and itch sensations [66]. The human MRGPRX1 was the first primate-specific MRGPR to be assigned to a bovine adrenal medulla 8–22 (BAM8–22) ligand. After, several other agonistic and antagonistic molecules were identified [58,67]. 

### 2.1. MRGPRX2 Ortholog in Mice and Expression Profile

The mouse genome possesses several family members of MRGPRs: MRGPRA (A1–A10), MRGPRB (B1–B5, B8), MRGPRC (C11), MRGPRD, MRGPRE, MRGPRF, MRGPRG, and MRGPRH genes [60,68]. MRGPRA1 and MRGPRB2 are considered as the mouse orthologs of human MRGPRX2 and share similar characteristics to MRGPRX2, such as exclusive expression on connective tissue MCs, DRGs and the ligand activation mechanism [69,70,71]. MRGPRA1 is expressed on DRGs and is activated by SP [60,71,72]. MRGPRB2 shows similar characteristics to MRGPRX2, such as exclusive expression on connective tissue MCs (not expressed on DRGs) and the ligand activation mechanism [60,71,72]. Immunochemistry studies have shown a high expression of MRGPRX2 in DRG neurons specifically, and the Cortistatin receptor is considered to be the most potent [73]. Additionally, MRGPRB2 is involved in neurogenic pain [74]; therefore, it can be speculated that MRGPRA1 is the major mouse ortholog that plays MRGPRX2’s role in DRG, whereas MRGPRB2 plays the role of MRGPRX2 in MCs.

MRGPRX2 is expressed on mast cells (MCTC but not in MCT subtypes) and dorsal root ganglions (DRGs) in humans and primates [45,73,75]. It is involved in host defense, pseudo-allergic reactions, non-histaminergic itch, periodontitis, neurogenic inflammation, and inflammatory pain [76,77,78]. MRGPRX2 binds with diverse agonists ranging from peptides to small molecules, insect venom chemical components, antimicrobial peptides, neuropeptides, and FDA-approved drugs [19]. MRGPRX3 and MRGPRX4 are expressed in human keratinocytes and are involved in cutaneous host defense response. There are not many studies reported on MRGPRX3–4 ligands; however, Toll-like receptor (TLR) ligands have been reported to upregulate the expression of MRGPRX3 and MRGPRX4 in human keratinocytes [79].

Although rodent MRGPRX2 (mouse MRGPRB2 and rat MRGPRB3) has similar characteristics such as selective expression in connective tissue MCs and similar ligand activation profiles [72], there is a considerable difference in efficacy of the agonists. For instance, the EC50 of several agonists for MRGPRB2 is higher than MRGPRX2 [72]. Additionally, the ligands’ selectivity in activation and inhibition of MRGPRB2/MRGPRX2 shows a considerable difference [71,80]. One possible reason can be the differences in the amino acid sequences of human MRGPRX2 and mouse MRGPRB2. Surprisingly, MRGPRX2/MRGPRB2 showed only ~53% overall sequence similarity, 34% N-terminal amino acids sequence similarity, and 47% C-terminal amino acids sequence similarity [19,45]. More recently, in 2020, dog MRGPRX2, which is the functional ortholog of human MRGPRX2, was identified [59]. Dog MRGPRX2 has shown selective expression in a limited number of skin tissues from the eyelid, abdomen, and cheek, which plays an essential role in drug-induced anaphylactoid reactions [59]. 

### 2.2. Structural Insights of MRGPRX2 and Signaling Pathway 

**Figure 2:**
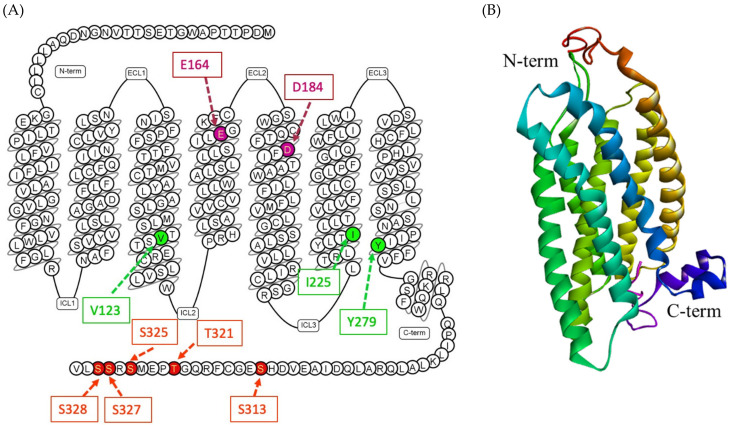
(**A**) Snake diagram of secondary structure of MRGPRX2 showing N-term, C-term, ECLs and ICLs, retrieved from GPCRdb (https://www.gpcrdb.org/, accessed on 15 April 2021) [90]. The amino acids involved in predicting the binding site of MRGPRX2 are E164 and D184 (pink color); amino acids involved in G protein coupling are V123, I225 and Y279 (green color); and amino acids involved in phosphorylation are S313, T321, S325, S327 and S328 (red color). (**B**) A predicted 3D homology model of MRGPRX2 showing N-term, C-term and seven transmembrane helix [77].

The human MRGPRX2 gene is a two-exon gene located on chromosome 11p15, 2036 bp in length, and encoding a protein with 330 amino acids [81,82,83]. The 3D structure of MRGPRX2 is yet to be identified; however, the homology modeling of the receptor revealed crucial structural information. The seven transmembrane (TM) bundles of MRGPRX2 are connected by three extracellular loops (ECL1, ECL2, and ECL3), and three intracellular loops (ICL1, ICL2, and ICL3). The N-terminus (N-term) of the extracellular (EC) domain is involved in ligand binding. The intracellular (IC) domain includes helix VIII and a C-terminal sequence is involved in G protein coupling and downstream signaling [84,85,86]. Recent experimental studies on MRGPRX2 structure biology have shown the crucial amino acids at the ECL and TM domain for binding and receptor activity [85,86,87,88] (Figure 2). The negatively charged residues, glutamic acid 164 (E164) and aspartic acid 184 (D184) at the ligand binding site of MRGPRX2, are crucial for binding several cationic ligands [69,87,88]. Since MRGPRX2 can be activated by a plethora of agonists, there might be more crucial amino acids that could be involved in ligand binding based on the nature of the ligand. In addition, there could be dual binding pockets for recognizing different ligands [69]. In TM domains, highly conserved class A GPCRs residues such as valine 123 (V123), isoleucine 225 (I225) and tyrosine 279 (Y279) are likely to participate in G protein coupling [86,89]. Furthermore, for receptor downstream signaling (G protein phosphorylation) at the carboxyl terminus of MRGPRX2, serine 313 (S313), threonine 321 (T321), serine 325 (S325), serine 327 (S327) and serine 328 (S328) are involved [86]. 

Recent research on naturally occurring MRGPRX2 missense variants and single-nucleotide polymorphisms (SNPs) revealed the possible mechanism of variation in the HSR in individuals [86,88]. The SNP may change the structure of MRGPRX2, which predisposes some individuals to MRGPRX2 hyperactivation. Thirty SNPs have been identified in the coding regions of human MRGPRX2, in which two of the most common SNPs are involved in phenotype changes. Amino acid substitution from asparagine 62 to tryptophan (N62T) and from asparagine 16 to histidine (N6H) may change the cytoplasmic domain 1 (CPD1) of MRGPRX2 and extracellular domain 1 (ECD1), respectively [88]. The four naturally occurring MRGPRX2 missense variants—glycine165 glutamic acid, aspartic acid184histidine, tryptophan243arginine and histidine259tyrosine (G165E, D184H, W243R, and H259Y)—failed to respond to MRGPRX2 agonists (SP, hemokinin-1, human β-defensin-3, and Icatibant) [88]. In another study, a similar group of potential missense variants was identified, with gain and loss of MRGPRX2-dependent SP-induced MC degranulation [86].

The missense variants valine123phenylalanine, arginine138cysteine arginine141cysteine and valine282methionine (V123F, R138C, R141C, and V282M) were identified as loss of function phenotypes for SP-induced mast cell activation and degranulation [86]. On the other hand, the missense variants serine325leucine and leucine329glutamine (S325L and L329Q) were identified as gain of function phenotypes for SP-induced mast cell activation and degranulation [86]. These findings have important clinical implications in identification of patients with listed SNPs. The individuals with loss of function MRGPRX2 variants can be resistant, while those with gain of function variants can be more susceptible to MRGPRX2 ligands. Prior identification of such variants may be helpful in categorizing susceptible individuals who can show drug-induced MC degranulation and HSRs. However, more studies are warranted to support these possibilities.

MRGPRX2 is a class A GPCR which activates the downstream signaling pathway via activation of trimeric G protein. Upon ligand binding to the N-terminal region of MRGPRX2, the Gαq protein is activated and leads to Ca^2+^ influx followed by MC degranulation [86,91]. Activation of the Gαq protein leads to activation of the phospholipase C-γ (PLCγ) pathway, which was inhibited by a PLCγ inhibitor U-73122 [48,92] Moreover, MRGPRX2 has also been reported to activate the PTx-sensitive Gαi pathway. PTX blocked the Ca^2+^ mobilization and MC degranulation activity of MRGPRX2 agonists such as compound 48/80 [93], SP [94], and Icatibant [95]. Interestingly, some MRGPRX2 agonists have shown a biased signaling mechanism via the beta-arrestin pathway [95]. It is of great interest to explore the beta-arrestin pathway of MRGPRX2, which causes biased signaling [87,95]. These findings suggested the involvement of Gαq, Gαi and beta-arrestin in MRGPRX2 signaling. MRGPRX2 activation can trigger several downstream signaling pathways, which contributes to ongoing allergic and inflammatory reactions [96]. MRGPRX2 agonists such as human beta defensins and LL-37 have been reported to activate the MAPK/ERK, p38, and JNK pathways and increased the release of IL-31, PGE2, and leukotriene C4 [96,97]. A recent study reported the involvement of the Nrf2 pathway in MRGPRX2-mediated MC degranulation and pseudo-allergic reactions [98]. Indeed, more studies are warranted to understand the downstream signaling pathways of MRGPRX2.

### 2.3. Diversity of MRGPRX2 Agonists 

**Figure 3:**
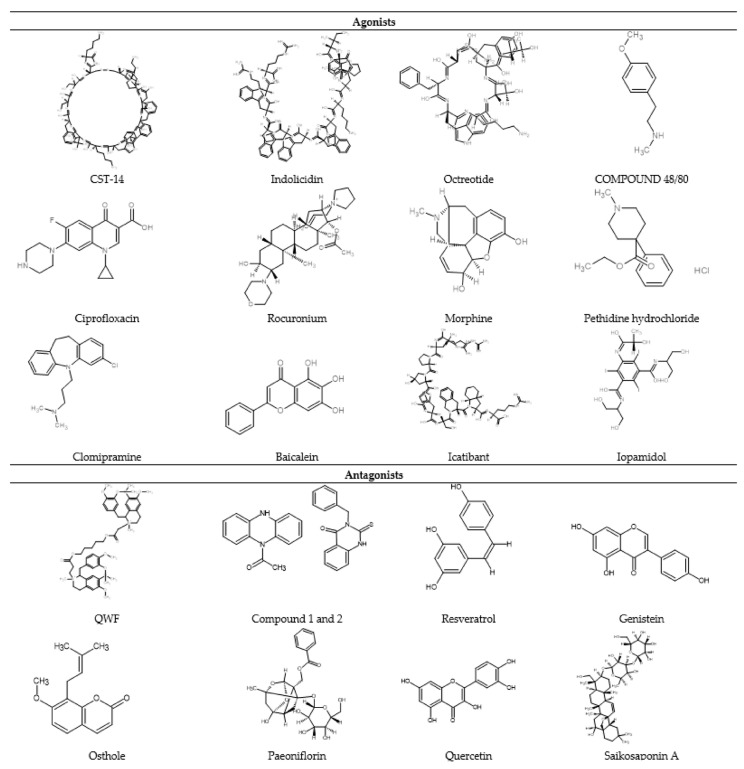
2D chemical structure of some MRGPRX2 agonists and antagonists.

A plethora of MC-degranulating compounds such as peptide toxin mastoparan, neuropeptides and compound 48/80 have long been known to induce MC degranulation via an unknown GPCR. Another mechanism of receptor-independent or direct activation of G proteins (Gαi2 and Gαi3) to induce downstream signaling for degranulation has also been reported [43,44]. In recent years, MRGPRX2 has been identified as the unknown GPCR which binds with several compounds and induces MC degranulation [27,45]. Unlike other GPCRs that have selective and limited agonists, MRGPRX2 is known to have many agonists, including cationic amphiphilic drugs, insect venom chemical components, antimicrobial peptides, secreted eosinophil products, neuropeptides, small compounds, and natural compounds (Figure 3). 

Moreover, MRGPRX2 has been reported as a low-affinity and low-selectivity receptor, which allows its interaction with diverse ligands (Figure 1). For ligand binding and G protein coupling, MRGPRX2 ligands use the conserved residues in their transmembrane (TM) domains and carboxyl-terminus Ser/Thr residues, respectively. In the first demonstration of non-IgE-mediated MC degranulation via MRGPRX2, the basic secretagogues such as peptides and compound 48/80 have shown a concentration -dependent increase in reporter gene expression in MRGPRX2-expressing PC12 cells and Ca^2+^ mobilization in MRGPRX2 expressing human embryonic kidney (HEK293) cells [45]. Host defense peptides (HDPs) such as LL-37 are potent MC chemoattractants and can induce degranulation [99]. HDPs modulate TLR4 on MCs and increase the expression of TLR4, which may enhance their ability to detect invading pathogens [100,101].

Epithelium and neutrophil-derived HDPs (hBDs and LL-37) have recently been reported to activate human MCs via MRGPRX2 [50,102,103]. This activation leads to MC degranulation but also the release of significantly less pro-inflammatory cytokines [56]. However, keratinocytes show a different response against LL-37 challenge, possibly via the activation of MRGPRX3/4. This activation leads to degranulation with a very high generation of pro-inflammatory cytokines and promotes wound healing [79,97]. Thus, the interaction between keratinocytes and mast cells via MRGPRX3/4 and MRGPRX2, respectively, has demonstrated a potential pathway involved in cutaneous host defense and wound healing. In addition, SP, a known endogenous ligand of MRGPRX2, has been shown to play a role in neurogenic inflammation and pain associated with wound healing by recruiting innate immune cells to the injury site [74]. In the following section, we have summarized the MRGPRX2 agonists into different groups according to their chemical nature such as peptides and non-peptides (drugs and natural compounds) (Table 1).

#### 2.3.1. Peptide Agonists

##### Cortistatin-14 (CST)

CST-14 was the first reported ligand for MRGPRX2, which increases the intracellular Ca^2+^ level without affecting the basal or forskolin-induced cyclic adenosine monophosphate (cAMP) levels [73]. This indicates the involvement of G protein pathways other than Gαs such as Gq or Gi protein in MRGPRX2 activation. Later, Gi and Gq G proteins were reported to be involved in the MRGPRX2 pathway [45,75]. CST-14 is a neuropeptide that possesses similar structural and biological activity to somatostatin-14 via binding to all somatostatin receptors with no selectivity for subtypes. However, some biological effects of CST-14, such as reducing the locomotor activity and inducing slow-wave sleep, distinguish it from somatostatin-14 and raise the possibility of a CST-specific receptor [73,117]. MRGPRX2 is a highly expressed GPCR in the DRG’s small-diameter neurons and is activated by CST-14 in a concentration-dependent manner [73]. Additionally, recent studies reported that CST-14 uses store-operated Ca^2+^ entry via Stromal interaction molecule 1 (stim1) for MRGPRX2-induced MC activation, which leads to MC degranulation, Ca^2+^ mobilization, and cytokine release in a concentration-dependent manner [104].

##### Substance P (SP)

SP is an inflammatory neuropeptide that exhibits a potent endogenous pruritogenic activity in humans and rodents. A well-known receptor for SP is the neurokinin-1 receptor (NK-1R). Several NK-1R pharmacological antagonists have been developed to block SP-induced itch, nociception, and inflammation in mouse models; however, these antagonists have shown mixed response in human trials. MRGPRs have been reported in primary sensory neurons and on nerve endings of dorsal root ganglia [70]. SP specifically activates human skin-derived cultured MCs through MRGPRX2, not through NK1R [46]. A recent study demonstrated the involvement of MRGPRA1 in sensory neurons rather than NK-1R in SP-induced itch response [70].

Interestingly, SP has shown more sensitivity to NK1R compared to MRGPRX2; the EC50 of SP for NK1R and MRGPRX2 is 0.5 nM and 100 nM, respectively [71]. It is also noteworthy that the receptor’s absolute number expressed on MC may not be the same. MRGPRX2 has shown a higher level of expression in the skin MC (SMC) transcriptome as compared to FcϵR1 [12]. SMCs expressed less NK1R than MRGPRX2, which can be a potential reason why SP binds and activates SMCs via MRGPRX2, not NK1R [118]. Evidence has been presented that suggests MRGPRX2 is an important target for treating MC-mediated pathological actions and histamine-independent itch [119].

##### Somatostatin

Somatostatin, or the growth hormone-inhibiting hormone, is a major neuropeptide widely distributed in the central nervous system and peripheral tissues [120]. There is limited evidence on MCs’ degranulation activity of somatostatin via MRGPRX2 activation [45,73].

##### Vasoactive Intestinal Polypeptide (VIP)

VIP is a neuropeptide that binds to VPAC (VPAC1 and 2) receptors and exhibits several biological activities, such as systemic vasodilation, hyperglycemia, smooth muscle relaxation, hormonal regulation, analgesia, and hyperthermia [121]. Human MCs have been reported to express surface VPAC2, which was increased via IgE-mediated human MC activation [42]. However, the MC activation mechanism of VIP is not mediated through VPAC2 but via an unknown GPCR [122]. The pioneer study by Tatemoto et al. reported MRGPRX2 as the GPCR responsible for VIP-induced concentration-dependent human MC degranulation [45].

##### Pituitary Adenylate Cyclase-Activating Polypeptide (PACAP)

PACAP is a homolog to VIP which exhibits multiple roles such as regulation of the metabolism, cardiovascular, endocrine, and immune systems [123]. Like VIP, PACAP has also been reported to activate MCs and release histamine, and this action is not mediated via VPAC2 [122]. PACAP is also a potent agonist for MRGPRX2 and causes concentration-dependent MC degranulation [45]

**Host defense peptides (HDPs**): HDPs such as cathelicidins and defensin are positively charged peptides that exhibit broad-spectrum antimicrobial activity via an MC–MRGPRX2-mediated mechanism [124]. 

##### Human Cathelicidins

Cathelicidins possess an N-terminal signal peptide and a C-terminal antimicrobial domain corresponding to the mature antibacterial peptide [125]. LL-37 is a potent cathelicidin reported to increase the expression of Toll-like receptor-4 on the MCs and induce MC degranulation [100,126,127]. Additionally, LL-37 has been reported to activate MCs via the pertussis toxin-sensitive G protein and phospholipase C-mediated signaling pathways [128]. The seminal work of Subramanian and coauthors solved the puzzle of GPCR involvement in MC activation. They reported MRGPRX2 to be an MC receptor which mediates several HDPs, including LL-37-induced MC degranulation, chemokine generation, and chemotaxis in the host cells [50,103]. Subramanian and coauthors elucidate the mechanism of MRGPRX2 in LL-37-induced MC degranulation via a battery of in vitro experiments such as hairpin RNA-mediated knockdown of MRGPRX2 in LAD-2 cells, degranulation assay, and Ca^2+^ flux assay [50].

##### β-Defensins

β-defensins are a family of mammalian cationic antimicrobial peptides (AMPs) known to play a major role in host defense [129]. Human β-defensin-2 has been reported to increase Ca^2+^ flux and histamine release in MCs via the Gq protein–phospholipase C signaling pathway [128,130]. In 2013, MRGPRX2 was reported as the MCs GPCR involved in human β-defensin-induced MC degranulation [103]. Human β-defensins-2 showed both in vitro rat peritoneal MC degranulation activity and increased vascular permeability in rats. Human MCs such as LAD-2 cells endogenously express MRGPRX2, while rat basophilic leukemia RBL-2H3 cells, and murine bone marrow mononuclear cells do not express MRGPRX2. Under human β-defensins-2 treatment, murine MCs did not show degranulation activity. However, upon ectopic expression of MRGPRX2, these cells responded to human β-defensins-2 and have demonstrated degranulation activity [103]. Furthermore, these finding are supported by silencing the expression of MRGPRX2 in human MCs that resulted in inhibition of human β-defensins-2 induced degranulation [103].

##### Indolicidin

Indolicidin is another AMP purified from the cytoplasmic granules of bovine neutrophils known to exhibit activity against Gram-positive bacteria, Gram-negative bacteria, and fungi [83]. Tatemoto et al. reported indolicidin as cationic AMPs, which increased Ca^2+^ influx in MRGPRX2-expressing cells and demonstrated a concentration-dependent degranulation of human MCs [45]. However, not many studies have been reported on the activity of indolicidin in inducing MRGPRX2 and its role in the immune response.

##### Angiogenic Peptide 30/5C (AG-30/5C)

AG-30/5C is an analog of AG-30 that can increase antimicrobial and wound healing activity [49]. A recent study reported the immunomodulatory activity and biased MRGPRX2 agonism of AG-30/5C [95]. Additionally, an unknown GPCR has been reported to be behind AG-30/5C-induced chemotaxis, cytokine and chemokine production [49]. Ganguly et al. reported MRGPRX2 as the GPCR on MCs, which caused Ca^2+^ flux and MC activation [95]. However, more studies are needed to understand its mechanism and role in antimicrobial and wound healing activities. 

##### AMPs Derived from Insulin-Like Growth Factor-Binding Protein 5 (AMP-IBP5)

AMP-IBP5 is derived from insulin-like growth factor-binding protein 5 (IGFBP-5) by serine proteases digestion [131]. Song et al. recently reported the mechanism of immunomodulatory wound healing through MCs–MRGPRX2 [48]. AMP-IBP5 has shown Ca^2+^ flux followed by MC degranulation and PGDs, and cytokines/chemokines release. In addition to antimicrobial activity, AMP-IBP5 showed immunomodulatory and wound healing activity via recruiting and activating MCs at the inflammation and wound site [48].

##### Proadrenomedullin N-terminal peptides (PAMP 20) 

PAMP-20 is a 20-amino acid peptide expressed in the adrenal medulla and showed a concentration-dependent histamine release activity from MCs [132]. PAMP 20 and its truncated analogs (9–20; also known as PAMP-12) have been reported as endogenous ligands for MRGPRX2 [75]. PAMP-20 and its truncated analogs are structurally similar to cortistatin, which might be one reason why they induce MRGPRX2 activation. Both cortistatin and PAMP bind at the same active site and may compete for the binding [105].

##### Mastoparan

Mastoparan is a wasp venom peptide toxin reported to show nonspecific secretagogue activity and releases histamine from MCs [133,134]. Human LAD2 MCs were treated with a graded concentration of mastoparan to evaluate MC degranulation and the release of inflammatory mediators such as TNF-α, PGD-2 and histamine [72]. Mastoparan showed concentration-dependent MC degranulation, and release of inflammatory mediators [72]. Furthermore, wild-type LAD2 MCs and MRGPRX2 siRNA LAD2 MCs were used to verify the MRGPRX2-dependent activity of mastoparan. Mastoparan at a concentration of 5 µg/mL has demonstrated MC degranulation activity in wild-type MCs, which was absent in MRGPRX2 siRNA MCs [72]. 

##### Eosinophil Granule Proteins

Human eosinophils granules proteins such as major basic protein-1 (MBP-1), major basic protein-2 (MBP-2), eosinophil peroxidase (EPO) and eosinophil cationic protein (ECP) have been well studied concerning their activity in viral defense and activation of effector cells [135,136]. Additionally, MBP, EPO, and ECP are known to activate MCs and release histamine via unknown receptors [106,137]. MRGPRX2 discovery on human MCs [45] solved the puzzle of unknown receptor and provided direct evidence of MBP, EPO, and ECP binding to MRGPRX2 and MC activation [46,106]. On the contrary, another eosinophil granule protein, eosinophil-derived neurotoxin (EDN), did not show MRGPRX2-dependent Ca^2+^ influx or histamine release from MCs [46]. It is worth noting that peptide MRGPRX2 agonists are rich in hydrophobic and cationic amino acids such as proline, phenylalanine, tryptophan, and arginine/lysine [138]. This contrary effect of EDN can be attributed to its lower levels of essential amino acids than other proteins such as MBP, EPO, and ECP [46].

##### Proteases

Proteases are known for hydrolysis of peptide bonds [139]. Recently, several proteases have been reported to activate MRGPRX2. For instance, cysteine endopeptidase cathepsin S [107] and mucunain [140] were reported to elicit allergic responses that activate MRGPRX2. Preclinical and clinical studies have suggested cathepsin S involvement in the pathogenesis of chronic inflammatory and pruritic skin diseases [141,142]. The chronic inflammatory and itch response of cathepsin S is attributed to both MRGPRX2 and PAR2 [107,108]. 

##### Gonadotropin-Releasing Hormone (GnRH) Receptor Agonist and Antagonist

GnRH receptor agonists such as sermorelin and octreotide have shown activation MCs via MRGPRX2. A study by McNeil et al. on potential MRGPRX2 agonists has demonstrated the agonistic potential of sermorelin and octreotide [72]. Both sermorelin and octreotide increased Ca^2+^ mobilization in MRGPRB2/MRGPRX2-transfected cells. Moreover, a GnRH receptor antagonist, cetrorelix, has been reported to increase Ca^2+^ mobilization in MRGPRB2/MRGPRX2 cells [72]. However, further evidence is warranted to establish the role of these peptides on MC–MRGPRX2 activation.

#### 2.3.2. Non-Peptide Agonist

Several non-peptide chemical compounds such as experimental drug compound 48/80, several classes of FDA-approved drugs, and natural compounds have been reported to cause allergic reactions via MRGPRX2-mediated MC activation (Table 1). The FDA-approved drugs include fluoroquinolone antibiotics, neuromuscular blocking agents (NMBAs), opiates, and antidepressants [143].

##### Compound 48/80 (C48/80)

Compound 48/80 is a synthetic condensation polymer of N-methyl-p-methoxy phenylethylamine and formaldehyde [144]. It has been used in experimental biology to study humans and rodents’ MC degranulation [144]. Recent studies have reported MC activation mechanisms via MRGPRX2 in different populations of MCs [45,72,109]. Compound 48/80 showed MC degranulation activity in differentiated MCs, mature MCs, LAD2 MCs, and CD341 cell-derived MCs, but failed to activate immature human MC (HMC-1) because of the lack of MRGPRX2 expression in HMC-1 cells [109]. MC activation was significantly reduced in MRGPRX2 siRNA (siMrgprX2-LAD2 cells) cells, which demonstrated the MRGPRX2-dependent activity of compound 48/80 [72]. Compound 48/80 is a commonly used MRGPRX2 agonist in studying MC biology, allergic pathways, and the non-IgE pathway, and for the evaluation of potential antagonists.

##### Fluoroquinolone Antibiotics

Fluoroquinolone antibiotics, such as ciprofloxacin, moxifloxacin, and norfloxacin, are commonly used broad-spectrum antibiotics [143,145]. In a recent case report, the authors reported an MRGPRX2-linked hypersensitivity reaction from a topical fluoroquinolone ophthalmic preparation [110]. Evidence has been reported on fluoroquinolone-induced MC activation via MRGPRX2 [72,111]. Ciprofloxacin increased MC degranulation and MRGPRX2-dependent Ca^2+^ release in a concentration-dependent manner [72]. Moreover, ex vivo stimulation of wild-type and MRGPRB2 mutant mice has demonstrated an MRGPRB2-dependent MC activation [72]. Contrary to this finding, one researcher found no activity of ciprofloxacin in an MRGPRX2-expressing stable cell line Ca^2+^ release assay up to a 100 μM concentration [87]. Possible reasons may be the differences in the ciprofloxacin concentration used, application type, or study design. In another study, several fluoroquinolone antibiotics were tested for MRGPRX2 activation potential via in vitro and in vivo mouse models [111]. Fluoroquinolone antibiotics have shown a concentration-dependent MC degranulation, Ca^2+^ release and increased vascular permeability through an MRGPRX2-dependent mechanism [111].

##### Neuromuscular Blocking Agents (NMBAs) 

The commonly used preoperative anesthetics, NMBAs, have been reported to present hypersensitivity allergic reactions. In a retrospective study, researchers reported succinylcholine as being a more allergic NMBA than atracurium during preoperative anesthesia [146]. Patients with rocuronium-induced allergic reaction showed negative specific IgE (sIgE) and basophil activation test (BAT) [147,148], which indicates a non-IgE-mediated pathway. Recently, several findings have demonstrated the mechanism and role of MRGPRX2 in NMBAs-induced preoperative pseudo-allergic reactions [72,85]. Rocuronium has shown concentration-dependent MC–MRGPRX2 activation of both murine and human MRGPRX2 [85]. Moreover, other NMBAs such as mivacurium and atracurium have also been reported to induce MC Ca^2+^ mobilization and degranulation, which was absent in MRGPRX2 knockdown MCs [72,112]. Mivacurium has demonstrated a concentration-dependent activation of MRGPRX2, MC degranulation, histamine, and inflammatory cytokine release [112]. Furthermore, mivacurium has shown increased paw extravasation and decreased body temperature in wild-type mice, while these effects were absent in MRGPRB2 knockdown mice [112]. However, more critical clinical data and experimental studies are needed to understand the mechanism of MRGPRX2-mediated pseudo-allergic reaction by NMBAs. 

##### Opioids and Their Derivatives

Opioids and their clinically used derivatives have long been known to cause MC degranulation [149,150]. Clinically used opioid derivatives, also known as opiates, have been used as positive controls in skin prick testing and intradermal allergic tests [151,152]. Opiates have been reported to activate MCs via MRGPRX2, which leads to activation of fibroblasts and intrathecal mass formation on chronic opiate infusion [114]. Morphine showed a concentration-dependent activation of MRGPRX2 and MC activation [114]. Recent studies revealed the involvement of MRGPRX2 as the preferential receptor for opioid allergic reactions [4,87]. Human SMCs that are known to predominately express MRGPRX2 have shown a degranulation response against morphine, while lung and heart MCs failed to respond [153]. Lansu et al. studied the dextro-enantiomers and N-methyl scaffolds of opioids (dextrorphan, dextrabenzylorpan and levorphanol) and several endogenous opioid ligands (dynorphin A(1–17), dynorphin A(1–13), and dynorphin A(1–9)) for MRGPRX2 activation activity [87]. These opioids ligands have demonstrated a concentration-dependent MRGPRX2 activation, Ca^2+^ mobilization and MC degranulation activity [87]. 

Moreover, an opioid analgesic, pethidine hydrochloride, was reported to increase MC degranulation and inflammatory cytokine release concentration dependently. In a recent study, two clinically used opioid receptor agonists, pethidine hydrochloride and fentanyl citrate, have been studied for MC activation and pseudo-allergic mechanism [113]. Pethidine hydrochloride has shown MRGPRX2-dependent MC degranulation and cytokine release activity, while fentanyl citrate did not show any activity [113]. This response of pethidine hydrochloride was dependent on MRGPRX2, which was evidenced by reduced MC degranulation and reduced inflammatory cytokine release in MRGPRX2 knockdown MCs [113]. This difference can be attributed to their difference in chemical structure.

##### Antidepressant Drugs

In a very recent study, Wolf et al. reported several cationic amphiphilic FDA-approved drugs that activate human and mouse MRGPRX2/MRGPRB2. Drugs such as clomipramine, paroxetine, and desipramine showed a concentration-dependent MRGPRX2/MRGPRB2 activation, MC degranulation, and scratching behavior in mice [115]. Moreover, the intradermal injection of clomipramine to a healthy human volunteer showed a typical wheal and flare reaction response and activated human SMCs [115].

##### Others

Drugs such as Icatibant (bradykinin B2 receptor antagonist), diagnostic agents such as iopamidol, and the antibiotic, vancomycin, have been reported to cause pseudo-allergic reactions, angioedema and serious hypersensitivity reactions at injection sites. These responses are attributed to MRGPRX2-mediated MC activation followed by MC degranulation and release of proinflammatory cytokines and histamine [72,154]. These responses are MRGPRX2-specific, as evidenced by the lack of MC degranulation or Ca^2+^ mobilization in MRGPRX2 knockdown MCs [72,95]. The glycopeptide antibiotic, vancomycin, causes a non-IgE-mediated hypersensitivity reaction known as red man syndrome. Recently, MRGPRX2 has been reported as a non-IgE mechanism of vancomycin-induced MC degranulation [155]. 

Moreover, several natural compounds that are used in traditional medicine systems have been reported to causes allergic responses in some individuals [156,157,158]. Some studies have recently shown the MRGPRX2-dependent MC activation and pseudo-allergic activity of these natural compounds such as baicalein and sinomenine. Sinomenine is a natural alkaloid that can induce MRGPRX2-dependent histamine release, Ca^2+^ mobilization, the release of MCP-1, IL-8, and MIP-1α, and tissue extravasation and paw edema in mice [92]. Another natural compound present in traditional Chinese medicine is baicalin, which has been reported to activate MCs via MRGPRX2 in a concentration-dependent manner. These pseudo-allergy reactions induced by baicalein were absent in MPGPRB2-knockout mice [116].

## 3. MRGPRX2 Unlocking the Puzzle of Non-IgE-Mediated Pseudo-Allergic Reaction

Allergic diseases are prevalent all over the world and are common in all age groups. For instance, in the United States, allergies are the sixth leading cause of chronic illness, affecting over 50 million Americans with a cost of 18 billion USD/year [159,160]. In Hong Kong, allergic diseases have been reported as fast-growing diseases with a high burden in pediatric populations [161,162]. Growing evidence on the high prevalence of allergic diseases and their adverse effect on public health highlights the need for novel therapeutic targets and drugs. As mentioned earlier, a plethora of drugs such as muscle relaxants, opioids, Icatibant and fluoroquinolones cause pseudo-allergic reactions via activating MCs through MRGPRX2, but not IgE. Additionally, accumulating evidence suggests the crucial role of MRGPRX2 in the neuroimmune interaction and the pathophysiology of allergic diseases such as chronic urticaria, allergic rhinitis, asthma, and food allergy [163]. 

MCs are the key player in allergic pathophysiology of allergic diseases, extending their role in neuroimmune crosstalk [164]. MCs reside in close proximity to peripheral nerve endings and have been demonstrated to interact with neuronal cells via MRGPRX2, which causes neurogenic inflammation and itch [57,74]. While IgE-FcεRI-mediated MC activation is an eminent and more studied mechanism in allergic diseases, recent studies demonstrated the crucial role of MRGPRX2 in the IgE-independent MC activation mechanism and allergic diseases. In clinical settings, several acute hypersensitivity reactions have been reported where the allergic component, IgE, was not detected in the serum; however, these reactions are precipitated by MC degranulation. For instance, in chronic idiopathic urticaria (CIU), despite comparable MC numbers, CIU patients demonstrated a strong immune response to intradermal SP and VIP injections via unknown precipitating factors (possibly MRGPRX2) [19,165,166]. 

Recently, a study reported a high expression of SMC MRGPRX2 in CIU patients compared to healthy SMCs, which solved the puzzle of unknown precipitating factors [46]. These studies are supported by several other studies on SP and hemokinin-1. Endogenous agonists such as SP and hemokinin-1 (HK-1) are known to be involved in causing atopic dermatitis and allergic asthma via activating neurokinin-1 receptor (NK-1R GPCR) [167]. However, recent studies have revealed the MRGPRX2-dependent MC activation mechanism by SP and HK-1 [74,168]. This effect was independent of NK-1R activation, indicating the crucial role of MRGRPX2 in the previously described effects of these neuropeptides on atopic dermatitis and allergic asthma. 

Vancomycin is a glycopeptide antibiotic widely used to treat methicillin-resistant staphylococci nosocomial infections [169]. The most common adverse effect of vancomycin is red man syndrome, which is IgE-independent and manifests via erythema of the head and upper torso, pruritus, urticaria, and hypotension [169]. Vancomycin has shown MRGPRX2 agonistic activity and activates MCs. Vancomycin increased MRGPRX2-dependent secondary messenger Ca^2+^ influx and MC degranulation [155]. 

Several other pseudo-allergic reactions elicited by MC mediators in the absence of IgE are injection-site reactions, and idiopathic MC activation in patients with mastocytosis. Interestingly, several of these patients were not sensitized to typical allergens and not in contact with a precipitating allergen [2,40]. The seminal work of McNeil et al. identified and characterized MRGPRX2 as a crucial MC receptor for pseudo-allergic drug reactions [72]. Commercial drugs commonly used in clinical settings such as Icatibant, cetrorelix, leuprolide, and sermorelin showed MRGPRX2-dependent activation of MCs. A common tetrahydroisoquinoline (THIQ) motif in intravenous small compound drugs such as neuromuscular blocking agents (NMBAs) and fluoroquinolones was identified. Most NMBAs (except succinylcholine) and fluoroquinolones (ciprofloxacin, moxifloxacin, levofloxacin, and ofloxacin) have been reported to cause pseudo-allergic reactions by activating MCs through MRGPRX2/MRGPRB2 [72]. Another study has reported the inhibition of atracurium (NMBA) and ciprofloxacin-induced human and mouse MC–MRGPRX2/MRGPRB2 activation by the NK-1R antagonist, QWF (glutaminyl-D-tryptophylphenylalanine) [71]. 

Recently, several perioperative drugs such as anesthetics, analgesics, NMBAs, iodinated contrast agents and antibiotics have been screened for MRGPRX2 MC activation [4]. Additionally, sera were collected from patients who had experienced an anaphylactoid reaction during anesthesia and were tested for MC degranulation activity. The sera from anaphylactoid reaction patients showed MC degranulation, compared to healthy patient sera. Moreover, upon MRGPRX2 silencing, MC degranulation activity was diminished significantly, which indicates this is an MRGPRX2-dependent activity. 

In a recent clinical study, a non-IgE-mediated pathway was identified in a patient who experienced severe pre-operative hypersensitivity to rocuronium [148]. Furthermore, genomic DNA sequence analysis revealed the presence of three missense mutations (M196I, L226P and L237P) in MRGPRX2’s fifth and sixth transmembrane domains. However, rocuronium did not induce degranulation in transiently MRGPRX2 transfected rat basophilic leukemia (RBL-2H3) cells [148]. This suggests a change in MRGPRX2 susceptibility in patients with MRGPRX2 mutation and a complex underlying mechanism. The follow-up study by Amponnawarat et al. showed that rocuronium has different affinity and potency to induce degranulation in murine and human MCs via MRGPRB2 and MRGPRX2, respectively. This indicates the crucial functional differences between human and mouse MRGPRX2/MRGPRB2 [85].

The role of mouse homolog MRGPRB2 in pseudo-allergic reactions, itch, and rosacea has been studied via several mouse models [72,170]. The commonly used mouse models are Evans blue extravasation and paw edema [72,77], the LL-37/compound 48/80 [171,172] induced itch model, and the LL-37 induced rosacea model [170,172]. The MRGPRB2 knockout model has provided direct evidence of the involvement of MRGPRB2/MRGPRX2 in pseudo allergies, itch, and rosacea. Recently, a humanized mouse model was developed via injecting GM-CSF and IL-3 plasmid into the NOD-scid IL2R-^γ−/−^ strain of mice [173]. This humanized model can be used as a robust animal model for in vivo screening of drugs [173].

In a recent experimental study, a typical iodinated radiocontrast medium which is widely used in clinical angiography has been reported to activate MRGPRX2 [3]. Iohexol-induced HSRs are common; however, its underlying mechanism was not clear. Iohexol has demonstrated pseudo-allergic responses in wild-type (WT) mice that are absent in KitW-sh/W-sh (MUT) mice. Furthermore, iohexol showed a Ca^2+^ influx response in MRGPRX2/MRGPRB2 transfected cells. The authors reported the crucial role of iodine in MRGPRX2 activation; after removal of iodine from iohexol, MC activation and local and systemic anaphylaxis were significantly reduced [3]. 

Several antidepressant drugs have been reported to cause pseudo-allergic reactions; however, their mechanisms remain unknown. In a recent study, a group of cationic amphiphilic antidepressant drugs including clomipramine, paroxetine, and desipramine has shown MRGPRX2 agonistic activity and induced scratching behavior in mice [115]. The clinically used antidepressant drugs, clomipramine, paroxetine, and desipramine, have demonstrated MRGPRX2/MRGPRB2-dependent MC activation and degranulation. Furthermore, intradermal injection of clomipramine to a human volunteer presented a wheal-and-flare reaction and activated human skin MCs. The EC50 of these drugs was in a micromolar range comparable to compound 48/80. Structure–activity relationship experiments may help in explaining drug-induced pruritus by similar cationic drugs [115].

## 4. Development of MRGPRX2 Antagonists

In recent years several molecules, including natural compounds and a few other molecules such as cytokines, small molecules, peptides, and DNA aptamers, have been reported to inhibit MRGPRX2-mediated pseudo-allergic reactions and inflammation (Figure 1). A detailed list of such MRGPRX2 antagonists is given in Table 2. However, low potency, a lack of studies including receptor binding studies, clinical studies, off-target effects, toxicity, in vivo dose–response curves, bioavailability and stability are the major concerns for these antagonists. Moreover, transgenic animals that express MRGPRX2 in MCs and independent validation by other groups are warranted. In the following sections, we summarized the molecules that have shown preclinical MRGPRX2 antagonistic/inhibitory activity. 

### 4.1. Pertussis Toxin (PTx)

PTx is secreted by *Bordetella pertussis* and exhibits a crucial role in bacterial colonization and in immunomodulation to evade innate or adaptive immunity [185]. PTx blocks Gαi signaling pathways and propagates Ca^2+^ mobilization and degranulation activity in a receptor-independent manner. Prior to MRGPRX2’s discovery, PTx-sensitive G protein (Gα_i_) was known to interact with cationic amphipathic peptides [186]. PTx is mostly used to understand the signaling pathway of GPCR and has been tested against a diverse range of agonists to verify its effect on MC degranulation. PTx antagonized the activity of HDP-induced degranulation in human MCs (endogenously expressing MRGPRX2) and MRGPRX2-transfected RBL-2H3 cells, while showing no effect on Ca^2+^ mobilization [50,103]. This indicates the dual MRGPRX2 signaling pathway (PTx-sensitive Gα_i_ and -insensitive Gα_q_ signaling pathways) induces MC degranulation. Moreover, HDPs have also been reported to cause the expression of the potent pruritic cytokine IL-31 via phosphatidylinositol 3-kinase (PI3K) and the p38, JNK, and ERK MAP kinases pathway in human MCs. This pathway was significantly blocked by PTx and MAP kinases inhibitors [97].

### 4.2. Tripeptide QWF (Gln-Trp-Phe)

QWF is a tripeptide composed of L-glutaminyl-L-tryptophyl-L-phenylalanine which showed dual antagonist activity against NK-1R and MRGPRs [71]. QWF showed substantial inhibition of SP-induced activation of MRGPRX2/MRGPRB2/MRGPRA1 and itch response in mice [70]. Moreover, QWF inhibited MC degranulation induced by compound 48/80, atracurium, and ciprofloxacin in human LAD2 MCs [71]. QWF is the only NK-1R antagonist that has been shown to inhibit MRGPRA1, MRGPRB2 and MRGPRX2 when compared with the known NK-1R antagonist aprepitant [71]. However, the plasma instability and lower bioavailability of QWF limits its therapeutic use [187]. Therefore, it is crucial to identify/develop an MRGPRX2 antagonist which offers both pharmacodynamic and pharmacokinetic advantages. 

### 4.3. Small Compound Antagonist

Thus far, there are only two small compound antagonists reported which have demonstrated significant inhibition of human MC degranulation and Ca^2+^ flux against SP and Icatibant [80]. However, these compounds failed to show similar activity in ex vivo mouse MCs [80]; one possible reason may be the difference between human and mouse MRGPRX2/MRGPRB2. 

### 4.4. Natural Compounds

Natural compounds are pharmacologically active chemicals obtained from naturally occurring living organisms. Medicinal plants, animals, and microorganism fermentation broths offer several unique and diverse chemical structures. Natural compounds have contributed to drug discovery and their development process. A vast range of FDA-approved drugs are based upon either natural compounds or derivatives of these. In recent years, several plant flavonoids, phenols, triterpenoid saponins, chalcones and glycosides have been reported to inhibit pseudo-allergic reactions by antagonizing MRGPRX2. In the following section, we have briefly outlined the natural compound antagonists. 

In a recent study, resveratrol showed inhibition of MRGPRX2-mediated MC activation via the Nrf2 pathway. Resveratrol inhibited compound 48/80-induced Ca^2+^ mobilization and MC degranulation. Additionally, resveratrol demonstrated attenuation of compound 48/80-induced hind paw extravasation, and systemic anaphylaxis in mouse models [98].

We recently identified a plant isoflavonoid, genistein, as a lead compound which showed MRGPRX2 antagonistic activity and a protective effect against compound 48/80-induced anaphylactoid shock [77]. Genistein attenuated MC degranulation, MRGPRX2 activation, and Ca^2+^ influx in a concentration-dependent manner. Moreover, genistein offset increased paw thickness and Evans blue extravasation in a mouse model of local anaphylactoid shock [77].

Osthole is a naturally occurring coumarin present in the fruits of *Cnidium monnieri* (L.) and demonstrated MRGPRX2 antagonistic activity. Osthole inhibited compound 48/80, SP, and LL-37-induced MC degranulation, Ca^2+^ mobilization, and chemokine/cytokine production in human LAD2 MCs. Additionally, osthole attenuated in vivo MC degranulation and prevented histological changes [170].

Flavanols such as kaempferol have been reported to inhibit both IgE-mediated [188] and non-IgE MRGPRX2-mediated allergic reactions [175]. Kaempferol dose-dependently decreased compound 48/80-induced mouse hind paw swelling, Evans blue extravasation, and MC degranulation. Additionally, kaempferol rehabilitated hypothermia and inhibited the release of histamine, tryptase, tumor necrosis factor-alpha, interleukin-8 and monocyte chemo-attractant protein-1 (MCP-1) [175].

A monoterpene glycoside, paeoniflorin, was recently reported to inhibit the effects of compound 48/80-induced pseudo-allergic reactions. Paeoniflorin attenuated MC degranulation, MRGPRX2 activation, and Ca^2+^ influx and downregulated the phosphorylation of key kinases such as PLCγ and MAPK/ERK. Paeoniflorin also inhibited Evans blue extravasation, paw inflammation, histamine, and chemokine release in a mouse model of pseudo-allergic reactions [176].

Shikonin is a plant naphthoquinone that demonstrated dose-dependent inhibitory activity against compound 48/80-induced pseudo-allergic reactions, systemic anaphylaxis, Ca^2+^ flux, and MC degranulation [174].

Plant alkaloids such as piperine demonstrated inhibition of MC degranulation, Ca^2+^ mobilization, cytokines and chemokines release. Piperine attenuated hind paw swelling and reversed hypothermia in a systemic anaphylaxis mouse model. Furthermore, piperine was tested for its binding affinity to MRGPRX2 via a chromatography method; piperine showed a competitive binding affinity with sinomenine hydrochloride (a plant-based agonist) [177].

Another plant flavonoid, quercetin, has been reported to antagonize MRGPRX2, inhibiting MC degranulation, Ca^2+^ response, and cytokine release. Furthermore, in an in vivo mouse model of local and systemic anaphylaxis, quercetin showed protective effects against inflammation and hypothermia [178].

Saikosaponin A is a triterpenoid saponin used widely in Chinese medicine that showed inhibition of compound 48/80-induced pseudo-allergic reactions via the MRGPRX2 pathway. Saikosaponin A attenuated compound 48/80-induced Ca^2+^ flux, MC degranulation and skin inflammation [179].

A plant pigment, hydroxysafflor yellow A, has also been reported to inhibit Ca^2+^ flux, MC degranulation and histamine release by compound 48/80, LL-37 and ciprofloxacin. In addition, hydroxysafflor yellow A attenuated compound 48/80-induced hind paw swelling and Evans blue extravasation in a mouse model [180]. 

Isoliquiritigenin is a plant-based chalcone that has been reported to inhibit compound 48/80-induced Ca^2+^ mobilization, MC degranulation, histamine, and cytokines release. Moreover, isoliquiritigenin showed attenuation of hind paw swelling, Evans blue extravasation, in vivo MC degranulation and tissue hemangiectasis in mouse models [181]. 

### 4.5. Others

Several other molecules are also reported to regulate or antagonize MRGPRX2 activation, such as DNA aptamers, cytokines, and stem cell factor (SCF). In a recent study, Suzuki et al. reported a novel DNA aptamer, X35, as an MRGPRX2 antagonist. DNA aptamer-X35 attenuated MC degranulation and histamine release induced by compound 48/80 and SP. Moreover, subcutaneous injection of aptamer-X35 decreased MRGPRX2-mediated anaphylactic shock [183].

Interleukin-33 (IL-33) is a tissue-derived nuclear cytokine that plays a crucial immune modulator role in allergic, fibrotic, and infectious diseases [189]. IL-33 has demonstrated to be an MC lineage-supportive and modulating cytokine; chronic exposure of skin MCs to IL-33 resulted in a significant reduction in MRGPRX2 and eliminated the pseudo-allergic/neurogenic route. In contrast, upon acute exposure (directly before stimulation with an agonist), IL-33-exposed MCs showed increased degranulation by MRGPRX2 ligands. This dual effect depends on the c-Jun N-terminal kinase (JNK) pathway [184]. 

Stem cell factor (SCF) is crucial in cell growth, survival, migration, and proliferation, depending on the cell type [190]. In MCs, besides growth and survival, SCF has been reported to promote degranulation via FcεRI [191]. In contrast, acute exposure of MCs to SCF decreased responsiveness to compound 48/80 and SP and potently inhibited MRGPRX2 pseudo-allergic degranulation. Moreover, SCF showed modulation of MRGPRX2-initiated cascade and receptor expression to inhibit the MRGPRX2 pseudo-allergic pathway [182].

## 5. Challenges and Future Directions

After its identification and characterization in 2006, MRGPRX2 is still in its infancy. MRGPRX2 is categorized as an orphan receptor, for which ligand–receptor binding studies and other studies (mentioned earlier in this paper) are lacking [63]. One major limitation is the unavailability of the 3D structure of MRGPRX2, which limits binding site information and experimental data interpretation [77]. However, molecular modeling experiments predicted the 3D structure of MRGPRX2 and identified a putative ligand–binding site and signaling region for MRGPRX2. Studies have shown the negatively charged amino acid residues E164 and D184 are crucial for binding. The single amino acid residue mutation in MRGPRX2 (Glu164Arg) prevents the interaction between the MRGPRX2 and several cationic agonists, including opioids and SP [69,87,88,192]. 

On the other hand, another MRGPRX2 agonist, cathelicidin LL-37, activates both wild and mutant receptors, indicating the possibility of the presence of different binding/activation sites for some ligands [69]. Accumulating evidence has suggested that MRGPRX2 is a universal and crucial receptor on MCs, and a variety of MRGPRX2 ligands have been reported. Ligands include not only drugs but also several endogenous peptides; however, the epidemic data have shown a lower frequency of MRGPRX2-mediated serious pseudo-allergic reactions. 

There are several reasons: First, the similarity of clinical signs and symptoms in IgE-mediated allergic reactions makes it very difficult to differentiate them from IgE-mediated reactions. In general, skin tests (prick/puncture and intracutaneous) are used to diagnose and confirm clinical sensitivity induced by aeroallergens, foods, and some drugs [193,194]. The percutaneous skin test is mainly used to diagnose the presence of IgE-mediated allergy. In addition, the quantification of drug-specific IgE antibodies is another primary in vitro test to determine immediate drug hypersensitivity reactions. Unfortunately, these tests failed to diagnose non-IgE-mediated allergic reactions in patients [195]. However, several other tests, such as in vitro testing, basophil activation tests (BAT), and drug provocation tests (DPT), are recommended for use in the diagnosis of non-IgE-mediated allergic reactions [196,197]. More rigorous, sensitive, and selective diagnosis methods are needed to identify IgE and non-IgE MRGPRX2-mediated allergic reactions. 

Second, the low-affinity and nonselective nature of MRGPRX2 makes it different from other GPCR. Therefore, a high concentration of drugs needed at the organs expressing MRGPRX2 (skin MCs), which is not very common at the therapeutic concentration used clinically. However, individuals with high expression or overactivity of MC-MRGPRX2 may be more susceptible to pseudo-allergic reactions. A recent population-based study on patients’ skin MCs has shown a significant difference in the MRGPRX2 ligand response. MRGPRX2 agonists, compound 48/80 and SP, demonstrated an MC activation response of between 3.1 and 52.4% and between 2.9 and 52.6%, respectively [191]. 

Third, the differences in MRGPRX2 signaling may decide the strength of response—stronger or weaker. The MRGPRX2 signaling pathway is comprised of Gi, Gq G-proteins, and β-arrestin [19,95]. Different G-protein activation patterns may exist in different cell types and the same cell type across subjects. This shows the possibility of inter-individual differences in signaling mechanism; however, further research studies are warranted to understand such effects. 

Fourth, several MC triggering factors such as drugs, food, and temperature may cause a cumulative strong MC activation [198]. For instance, during anesthesia, patients are exposed to several factors such as analgesics (opioids and NSAID), NMBA, temperature, etc., which may explain more reports of pseudo-allergic reactions during anesthesia in certain patients. However, more clinical and preclinical studies are needed to establish the role of MC triggering factors in MC MRGRPX2-mediated pseudo-allergic reactions. 

Another challenge is the lack of translatability of preclinical findings to clinical data or vice versa. For example, the binding affinity and response of MRGPRX2 ligands to human MCs (MRGPRX2) and mouse MCs (MRGPRB2) is different [80,199]. The difference in the sequence similarity of MRGPRX2 and MRGPRB2 has been discussed earlier in the text. These species-specific differences in binding affinity and efficacy in mice limit our ability to study the underlying mechanisms of pseudo-allergic reactions and further develop therapeutic drugs. In a recent study, the authors tried to overcome this issue by developing a humanized mouse model for MC–MRGPRX2-mediated cutaneous adverse drug reactions [173]; however, the inherent disadvantage [200] of humanized mouse models and cost are the limiting factors. 

All these findings suggest the great potential MRGPRX2 has in understanding MC biology. Overall, from all the available evidence, we can conclude that MRGPRX2 solves the puzzle of non-IgE-mediated pseudo-allergic reactions. Moreover, developing a specific MRGPRX2 antagonist could provide an in-depth understanding of MRGPRX2’s mechanism and a novel approach for the treatment of pseudo-allergic reactions.

## Figures and Tables

**Table 1:** Summary of the MRGPRX2/MRGPRB2 agonists (peptides, non-peptides and natural compounds).

SN	Agonist	Major Findings	References
**Peptide Agonist**			
1	CST-14	MRGPRX2-dependent MC activation, degranulation and cytokine release Increased Ca^2+^ mobilization Involvement of store-operated Ca^2+^ entry via stim1	[72,75,104]
2	Substance P	Presence of MRGPRs in primary sensory neurons and on nerve endings of dorsal root ganglion MRGPRX2-dependent activation of human skin-derived cultured MCs MRGPRX2-dependent itch response	[46,67,70]
3	Somatostatin	MRGPRX2-dependent MC degranulation	[45,73]
4	PACAP	MRGPRX2-dependent MC degranulation	[45]
5	VIP	MRGPRX2-dependent MC degranulation	[45]
6	LL-37	MRGPRX2-dependent LL-37 induces MC degranulation, chemokine generation, and chemotaxis Increased Ca^2+^ mobilization	[50,103]
7	β-defensins	Identification of MRGPRX2 in human β-defensin-induced MC degranulation Increased vascular permeability in rats	[103]
8	Indolicidin	Increased Ca^2+^ influx in MRGPRX2-expressing cells Dose-dependent degranulation of human MCs	[45]
9	PAMP 20	Identified as an endogenous ligand for MRGPRX2 Similar binding site as of Cortistatin	[75,105]
10	Mastoparan	Concentration-dependent MRGPRX2 activation Concentration-dependent MC degranulation and inflammatory mediators release activity	[72,91]
11	Human eosinophil granules	MRGPRX2-dependent activation of human MCs Identified major basic protein-1 (MBP-1) and major basic protein-2 (MBP-2), eosinophil peroxidase (EPO) and eosinophil cationic protein (ECP) as MRGPRX2 agonists	[45,46,106]
12	Proteases	Identified cysteine endopeptidase cathepsin S and mucunain allergic response via MRGPRX2 MRGPRX2-dependent chronic inflammation and itch response	[107,108]
13	AG-30/5C	Identified AG-30/5C as biased MRGPRX2 agonist Induction of chemotaxis and cytokine production via MRGPRX2-mediated MC activation	[95]
14	AMP-IBP5	Identified AMP-IBP5 as MRGPRX2 agonist Role in wound healing through MCs–MRGPRX2 AMP-IBP5 shown Ca^2+^ flux followed by MC degranulation and release PGDs, and cytokines/chemokines.	[48]
15	GnRHR agonist and antagonist	Sermorelin and octreotide as MRGPRX2 agonist Increased Ca^2+^ mobilization in MRGPRX2-transfected cells	[72]
**Non-peptide agonist**			
1	Compound 48/80	MRGPRX2-dependent MC activation of different population of MCs Increased Ca^2+^ mobilization, and cytokine release	[45,72,109]
2	Fluoroquinolone antibiotics (ciprofloxacin)	MRGPRX2-dependent hypersensitivity reaction by topical fluoroquinolone ophthalmic preparation Concentration-dependent MC degranulation, and MRGPRX2-dependent Ca^2+^ release by ciprofloxacin Dose dependent MC degranulation, Ca^2+^ release and increased vascular permeability	[72,110,111]
3	NMBAs (rocuronium, mivacurium, and atracurium)	Rocuronium-induced non-IgE-mediated allergic reaction clinically Concentration-dependent MCs–MRGPRX2/MRGPRB2 by rocuronium MC Ca^2+^ mobilization and degranulation concentration-dependent activation of MRGPRX2, MC degranulation, histamine and inflammatory cytokine release by mivacurium Increased paw extravasation and decreased body temperature by mivacurium	[72,85,112]
4	Opioids (morphine, pethidine chloride, dextrorphan, levorphanol)	MRGPRX2-mediated MC activation and intrathecal mass formation. Concentration-dependent MRGPRX2 activation and MC activity of morphine Dextro-enantiomers and N-methyl scaffolds of opioids and endogenous opioid ligands activates MCs via MRGPRX2 Increased Ca^2+^ mobilization and MC degranulation activity Concentration-dependent human MC degranulation and inflammatory cytokine release by pethidine hydrocholoride	[87,113,114]
5	Antidepressant drugs (clomipramine paroxetine and desipramine	Concentration-dependent activation of MRGPRX2/MRGPRB2 and MC activation Induced scratch behavior in mice Clomipramine showed wheal-and-flare reaction on intradermal injection and activated human skin MCs	[115]
6	Others (Icatibant, iopamidol, vancomycin, baicalein, and sinomemnin)	MRGPRX2/MRGPRB2 activation MC degranulation and Ca^2+^ mobilization Induced tissue extravasation and cytokine release	[72,92,95,116]

**Table 2:** Summary of the MRGPRX2/MRGPRB2 antagonists (peptides, small compounds and natural compounds).

S.N.	Antagonist	Major findings	References
1	Pertussis Toxin (PTx)	Decreased HDP-induced MC degranulation No effect on Ca^2+^ mobilization HDPs induced expression of IL-31 via the PI3K-MAP kinase pathway	[50,97]
2	Peptide QWF	Inhibited C48/80-, atracurium-, and ciprofloxacin-induced MC degranulation Inhibited SP-induced itch response	[70]
3	Small compounds (compound 1 and 2)	In vitro inhibition of SP- and Icatibant-induced MC degranulation and Ca^2+^ mobilization	[80]
**Natural compounds**			
4	Resveratrol	Inhibited C48/80-induced Ca^2+^ mobilization and MC degranulation via Nrf2 pathway In vivo inhibition of local and systemic anaphylaxis	[98]
5	Genistein	Inhibited MC degranulation, MRGPRX2 activation and Ca^2+^ mobilization In vivo inhibition of local anaphylaxis in mice	[77]
6	Shikonin	Inhibited MC degranulation and Ca^2+^ mobilization Inhibited C48/80-induced pseudo-allergic reactions and systemic anaphylaxis	[174]
7	Osthole	Inhibited SP-, C48/80- and LL-37-induced MC degranulation and Ca^2+^ mobilization Inhibited in vivo MC degranulation	[170]
8	Kaempferol	Inhibited C48/80-induced hind paw swelling, Evans blue extravasation and MC degranulation Inhibited the release of histamine and cytokines	[175]
9	Paeoniflorin	Inhibited MC degranulation, MRGPRX2 activation and Ca^2+^ mobilization Downregulated PLCγ and MAPK/ERK	[176]
10	Piperine	Inhibited MC degranulation, and Ca^2+^ mobilization Showed binding affinity with MRGPRX2 via chromatography	[177]
12	Quercetin	Inhibited MC degranulation, MRGPRX2 activation and Ca^2+^ mobilization Inhibited cytokine release Inhibited C48/80 induced pseudo-allergic reactions and systemic anaphylaxis	[178]
12	Saikosaponin A	Inhibited MC degranulation and Ca^2+^ mobilization Inhibited C48/80-induced skin inflammation	[179]
13	Hydroxysafflor yellow A	Inhibited C48/80-, LL-37- and ciprofloxacin-induced MC degranulation Inhibited C48/80-induced hind paw swelling and Evans blue extravasation	[180]
14	Isoliquiritigenin	Inhibited C48/80-induced Ca^2+^ mobilization, MC degranulation, histamine and cytokines release. Attenuated hind paw swelling, and Evan blue extravasation	[181]
15	Others (DNA aptamer, cytokines, and stem cell factor)	DNA aptamer inhibited C48/80- and SP-induced histamine release and MC degranulation. Dual effect of IL-33 on MC degranulation and MRGPRX2 activation Acute exposure of stem cell factor inhibited MRGPRX2 activation and MC degranulation	[182,183,184]

## Data Availability

Not applicable.
